# First Data on *Campylobacter* spp. Presence in Shellfish in Croatia

**DOI:** 10.3390/pathogens11080943

**Published:** 2022-08-19

**Authors:** Luka Jurinović, Biljana Ječmenica, Natalija Džafić, Diana Brlek Gorski, Borka Šimpraga, Fani Krstulović, Tajana Amšel Zelenika, Andrea Humski

**Affiliations:** 1 Croatian Veterinary Institute, Branch Poultry Centre, Heinzelova Str. 55, 10000 Zagreb, Croatia; 2 Croatian Veterinary Institute, Branch Veterinary Institute Rijeka, Podmurvice 29, 51000 Rijeka, Croatia; 3 Croatian Institute of Public Health, Rockefeller Str. 7, 10000 Zagreb, Croatia; 4 Croatian Veterinary Institute, Savska Str. 143, 10000 Zagreb, Croatia

**Keywords:** *Campylobacter jejuni*, *Campylobacter lari*, bivalve molluscs, MLST

## Abstract

This study aimed to assess the presence of thermotolerant *Campylobacter* spp., as one of the most important foodborne zoonotic pathogens, in three shellfish species: mussels (*Mytilus galloprovincialis*), oysters (*Ostrea edulis*) and queen scallops (*Aequipecten opercularis*). The samples were collected from nine locations in the Istrian aquatory, Croatia. Isolation of *Campylobacter* was done according to standard ISO method, and species were identified using multiplex PCR. Isolates identified as *C. jejuni* and *C. lari* were genotyped using multilocus sequence typing (MLST) to determine the potential source of contamination. Among 108 examined samples of bivalve molluscs, mussels dominated and were the only ones found positive for the presence of *Campylobacter* (25.6%). In total, 19 *C. lari* and 1 *C. jejuni* strains were isolated. *C. lari* isolates found in this study belong to 13 sequence types (STs), and 9 of them are newly described in this paper. Two out of the four previously described *C. lari* STs that were found in this study were previously found in human stool. The only *C. jejuni* isolate was found to be sequence type 1268, which belongs to ST-1275 clonal complex that is almost exclusively found in seabirds and can sporadically cause infection in humans. Regarding the obtained results, introducing surveillance of thermotolerant *Campylobacter* in shellfish in the Republic of Croatia is advised as an improvement for public health safety.

## 1. Introduction

In the Republic of Croatia, shellfish are a traditional part of the human diet and an important source of nutrients, especially easy-to-digest proteins. However, from a food safety viewpoint, shellfish are also a known cause of various toxoinfections due to the usual way of consuming them raw or undercooked. In fact, in the EU throughout 2020, there were 142 foodborne outbreaks due to “crustaceans, shellfish, molluscs and products thereof” [1].

In their natural filter-feeding process, shellfish can accumulate and concentrate various pathogenic microorganisms present in the surrounding water, including bacteria of the genus *Campylobacter* [2,3,4,5,6].

Thermotolerant *Campylobacter* spp., in particular *C. jejuni* and *C. coli*, and to a lesser extent *C. lari* and *C. upsaliensis*, are the causative agents of most cases of human campylobacteriosis. The main reservoirs of *C. jejuni*, *C. coli* and *C. lari* are considered to be poultry and cattle, poultry and pigs, and wild birds (especially gulls and shorebirds), respectively [7,8]. Due to *Campylobacter* specific tropism for the digestive system of animals along with the asymptomatic presence in the intestinal tract, soils and environmental waters contaminated with animal excretions are frequently contaminated with *Campylobacter* as well. Runoffs and watersheds from such contaminated areas have a large impact on the microbiological quality of the coastal waters nearby, increasing the possibility for the presence of pathogenic microorganisms as *Campylobacter*, as well for the outbreaks of campylobacteriosis after consumption shellfish grown in an environment polluted in such a way [9,10,11,12,13,14].

In the Republic of Croatia, campylobacteriosis has been a notifiable disease since 2007, and it has been the leading zoonosis in the country, with 1082 cases reported in 2020 [1]. According to epidemiological data for 2020, human campylobacteriosis in Croatia was attributed to the poultry reservoir as a whole, mainly caused by *C. jejuni* (72%), followed by *C. coli* (19%), while the remaining 9% were reported as *Campylobacter* spp. [15]. In support of this, a recent Croatian study reported that more than 30% of the neck skins of broiler carcasses are *Campylobacter*-positive with a high number of units above the limit of 1000 CFU/g. That study covered four Croatian regions, including the Northern Littoral where the Istrian peninsula is located, and where the percentage of samples that exceed the limit was among highest (32%) [16].

Although the EU legal framework [17,18] lays down the requirements that bivalve molluscs must meet at the time of sale (live or properly processed; of impeccable sensory characteristics; within permitted limits for the presence of biotoxins and *Escherichia coli*), studies have shown that *E. coli*, as a basic indicator of shellfish contamination, does not correlate with the presence of other microorganisms, especially pathogenic viruses and bacteria such as *Campylobacter*, *Vibrio* and *Salmonella* [19,20,21].

The aim of this study was to assess the presence of thermotolerant *Campylobacter* species in shellfish production and harvesting areas of the Istrian aquatory, regarding the growing conditions (in terms of location and sea temperature) and shellfish species, as factors influencing increased public health risk when consuming shellfish.

## 2. Results

A total of 108 samples of shellfish were investigated in the period from August to October 2021. The most numerous species of shellfish examined were mussels 72.2% (*n* = 78), followed by scallops 25.0% (*n* = 27) and oysters 2.8% (*n* = 3) (Table 1).

The presence of *Campylobacter* spp. was detected in 18.5% (*n* = 20) of all examined samples, and in 25.6% of tested mussel samples (*n* = 78). *C. lari* was the most frequently isolated species with 95% (*n* = 19) of samples positive for this species versus 5% (*n* = 1) for *C. jejuni.*

*Campylobacter* spp. were more frequently isolated in mussels sampled at the west coast (*n* = 17), of which, regarding the highest number of positive samples, stand out locations L-I (*n* = 7), L-IV (*n* = 5) and L-VI (*n* = 4). Similar frequency of *Campylobacter* positive samples at the West coast of the peninsula was observed during all of the months included in the study, with six positive each in August and September, and five of them in October (Table 2).

At the locations of the east coast (L-III and L-V), *Campylobacter* spp. were detected in three samples collected at the beginning of October.

The majority of *Campylobacter* spp., 85% of them (*n* = 17), were detected when the sea temperature was between 20 °C and 27 °C. The lowest sea temperature when *Campylobacter* positive sample has been collected, was 18 °C at location L-I on the West coast.

Multilocus sequence typing (MLST) was done on all isolates, and all of them gave full sequence type (ST) sequences. The only *C. jejuni* isolate from the present study was found to be of the ST 1268, which belongs to the ST-1275 clonal complex (CC). A total of 19 *C. lari* isolates yielded 13 different STs, and 9 of them are newly described (STs 300, 301, 302, 303, 304, 305, 307, 308 and 311). Additionally, ten new alleles are described in the present study (*adk* 147; *atpA* 153; *glnA* 122; *pgi* 186, 187, 188 and 189; *pgm* 173 and 174; *tkt* 164) (Table 3). All the *C. lari* STs were singletones (not matching any CCs).

Sea temperatures, measured during the sampling months, are shown in Table 2.

## 3. Discussion

In the period from August to October 2021, a total of 108 bivalve molluscs sampled at nine locations around the Istrian peninsula in the northern part of Adriatic Sea were tested for the presence of *Campylobacter* spp. These months were selected because it is the peak of the tourist season when increased demand for shellfish consumption is common.

In this study, *Campylobacter* spp. were isolated only from mussels. There are some studies that report more frequent finding of *Campylobacter*, as well the other pathogenic microorganisms (as *Salmonella* spp., *Vibrio* spp., human Noroviruses), in mussels rather than in other shellfish species due to their higher ability to concentrate bacteria during the seawater filtration process [19,22]. Whether the absence of *Campylobacter* detection in the remaining two species of bivalve molluscs investigated in this study could be attributed to the small number examined, or to their species, requires further research.

Depending on the sampling location, the presence of *Campylobacter* spp. varied from 0% in harvesting areas (L-VII to L-IX), to 25.6% on average and the highest 46.2% in production areas (L-I to L-VI). Out of all the investigated production areas, three locations (L-I, L-IV and L-VI) in the bays that go deep into the mainland stands out in terms of the overall number of *Campylobacter* spp. positive samples (75%). Such findings at these locations during the tourist season can be explained by a greater human impact on the whole—from urban and agricultural runoffs located upstream of coastal waters, through ballast waters of the ships to the presence of numerous tourists and their pets. However, the far greater possibility of such a finding could be due to contamination with faeces of seabirds staying on buoys for mussel farming [21,23]. Seabirds, especially gulls are known to carry *C. lari*. Our previous study showed that 11.3% of gulls caught on a Zagreb rubbish tip were positive for *C. lari* [24]. Most of them (15/19) were isolated from Yellow-legged Gull, *Larus michahellis*, (unpublished data), which is the most abundant breeding species of gulls in the Adriatic Sea [25].

The results of our study support the last statement as *C. lari* was the most commonly identified species with 24.4% versus 1.3% for *C. jejuni* of mussel samples investigated for the presence of *Campylobacter.* These findings are consistent with those reported by Rince et al. [20] where *C. lari* was the most frequently isolated species in shellfish, with 26.4% of samples positive for this species versus 0.8% for *C. jejuni*. Similarly, Wilson and Moore [19], in shellfish sampled in the UK, reported 2%, 8% and 24% contamination rates for *C. jejuni*, *C. coli* and *C. lari*, respectively. Contrary to the *Campylobacter* presence rates found in the present study, Lozano-León et al. [26] reported that in mussels harvested in Galicia, NW Spain, only 8% of tested samples (*n* = 91) were positive, with all isolates identified as *C. lari*. The dominance of *C. lari* in shellfish due to its better survival in the aquatic environment and higher salt-tolerance regarding to *C. jejuni* and *C. coli* was also reported in earlier studies [8,19,27,28].

The Adriatic is a sea of shallow depth, whose physical properties, in addition to the atmosphere, inland waters and interaction with the Ionian Sea, are significantly influenced by the topography of the basin. The general surface current flows in a counter clockwise direction from the Gate of Otranto along the east coast to the north and flows along the Italian coast to the south. The surface temperature of the Adriatic is the lowest in its northern part. During the year it is lowest in February and March, and highest in August [29]. Several studies on the influence of seawater temperature seasonality on the presence of *Campylobacter* show more frequent incidence in autumn and winter when seawater temperatures are below 15 °C, than in the summer months when the temperature values are higher, suggesting that *Campylobacter* may survive more easily under cold conditions [19,20,30,31]. In the present study, average sea temperature values recorded on the day of collecting the *Campylobacter*-positive shellfish samples ranged from 25.8 °C in August to 19.6 °C in October (Table 2), which is opposite to the findings of Wilson and Moore [19] who established less than 6% of shellfish examined between May and August *(n* = 162) containing *Campylobacter*. The results of our study are gained throughout the middle of summer and beginning of the autumn season, when the sea temperatures are expectedly higher. Further studies will reveal how much influence season and water temperature has on the occurrence of *Campylobacter*, considering the mentioned specifics of the Adriatic Sea.

The only *C. jejuni* isolate from the present study was genotyped using MLST and was found to be of the sequence type 1268, which belongs to the ST-1275 clonal complex (CC). Isolates belonging to this CC are almost exclusively isolated from seabirds (mostly gulls), shellfish and environmental waters, and sporadically from human stool [24,32]. This particular ST was found in American herring gulls (*Larus smithsonianus*), Black-headed gulls (*L. ridibundus*) from Sweden and Croatia, environmental waters from the Netherlands and Canada, goose from the USA and human stool from the UK.

In the PubMLST [32] online database (accessed 1 July 2022), there are 145 isolates of *C. lari* originating from shellfish. Most of them are from France (*n* = 114), then Croatia (*n* = 19), Germany (*n* = 7) and Denmark (*n* = 4). Just six isolates from the present study belong to known STs. All of these STs were previously found only in France; ST 76 in one isolate from human stool, ST 77 in three human stool isolates and one cockles isolate, ST 82 in one isolate from gulls (species not listed) and ST 223 in one isolate from mussels. In our previous study (unpublished data), we found STs 77 and 223 in Yellow-legged Gulls, *Larus michahellis*, feeding on a Zagreb city rubbish dump. These data also support the above statement about seabirds’ and humans’ impact on microflora found in bivalve molluscs.

The obtained data are the first that signify the presence of pathogenic *Campylobacter* species in shellfish from the production areas on the Croatian coast of the Adriatic Sea. As studies show that the occurrence of these bacterial species does not correlate with the values of *E. coli* bacteria presence [19,20,21], on which depuration measures of fresh bivalve molluscs are determined, it is to be assumed how bivalve molluscs contaminated with *Campylobacter* spp. are placed on the market.

Although all *Campylobacter*-positive samples have been found in mussels, future research on a bigger number of samples of different species of bivalve molluscs from all production and harvesting areas along the Croatian coast will indicate whether only mussels can be selected as an indicator species to monitor the occurrence of *Campylobacter* spp.

Even though campylobacteriosis caused by seafood, i.e., shellfish, has not been recorded in Croatia, the occurrence of sporadic cases and unrecognized cases of disease associated with the consumption of these products cannot be excluded. Additionally, a finding of *C. lari* isolates belonging to STs that are known to infect humans, is a good cause to monitor these bacteria in shellfish populations.

Thus, based on our results, an improvement of public health safety can be advised by introducing continuous surveillance of *Campylobacter* spp. in shellfish, by using whole genome sequencing to determine a linkage to its source.

## 4. Materials and Methods

**Figure 1:**
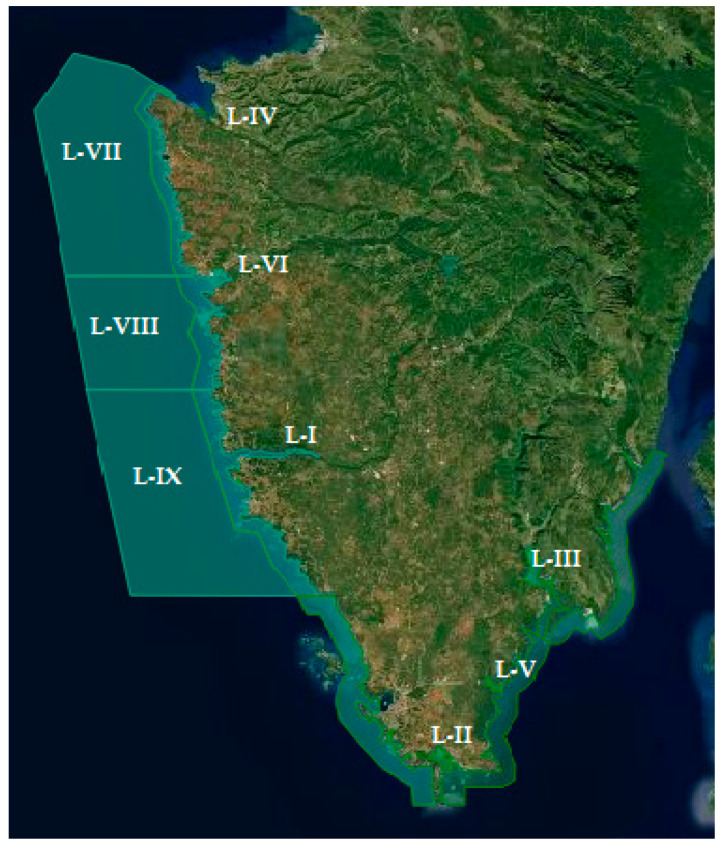
Geographical position of sampling locations.

The study of the presence of thermotolerant *Campylobacter* spp. included samples of the most economically important species of live bivalve molluscs: mussels (*Mytilus galloprovincialis*), oysters (*Ostrea edulis*) and queen scallops (*Aequipecten opercularis*) cultivated in the Istrian county (North Adriatic Sea) (Figure 1).

Area boundaries, reference types of microbiological contamination monitoring and geographical sampling points are defined for each production area (Figure 1) [33]. In the Istrian aquatory, shellfish were sampled on a weekly basis at six production areas where molluscs and oysters are farming (locations L-1 to L-VI), and three points for queen scallops harvesting (locations L-VII to L-IX). Production areas are located in gulfs, some of which are deeply indented into the mainland, such as location L-I and L-III, while locations L-II, L-IV, L-V and L-VI are more exposed to the natural influence of the open sea. Only two sampling locations are situated on the eastern coast of the Istrian peninsula (L-III, L-V), while other are placed on its western side. Data on the sea temperature was obtained from the Croatian Metereological and Hydrological Service and it was measured at permanent measurement points, at a depth of 30 cm [33].

After collecting directly from the water, the samples were transported in cool boxes at 4–8 °C and examined within maximum of 24 h from the time of sampling [33].

The detection of *Campylobacter* spp. was done according to the standard ISO 10272-1:2017 method [34]. Each laboratory sample comprised individuals within the normal commercial size and was pooled of ten animals. When delivered to the laboratory, the shellfish were washed with clean, tap water, and opened under aseptic conditions. The test portion (shellfish flesh and intravalvular liquid in the amount of 25 g) was added to the liquid enrichment medium (Bolton broth; Oxoid, UK). After incubation in a microaerobic atmosphere (CampyGen; Oxoid, UK) at 37 °C for 4–6 h and then at 41.5 °C for 44 h, a loopful (10 µL) of the enrichment cultures were streaked at the surface of each of the two selective plating mediums, modified Charcoal Cefoperozone Deoxycholate agar (mCCD agar; Oxoid, UK) and CampyFood ID agar (CFA; BioMerieux, France). The selective solid media were incubated at 41.5 °C in a microaerobic atmosphere and examined after 44 h to detect the presence of suspect *Campylobacter* colonies. All of presumptive *Campylobacter* colonies were examined for morphology and motility and sub-cultured on a Columbia agar (Oxoid, UK) with 5% sheep blood (BioGnost, Zagreb, Croatia) for further confirmation (microscopic verification of the characteristic morphology and motility, detection of oxidase activity, aerobic growth test at 25 °C).

Bacterial DNA was extracted by boiling a loopful of pure bacterial culture in 100 μL of PCR-grade water for 20 min and centrifuging at 14,000 rpm for a min. Species determination was done using multiplex PCR [35]. Multi-locus sequence typing (MLST) for *C. jejuni* was performed according to Dingle et al. [36] and for *C. lari* according to Miller et al. [37]. PCR products were sequenced at Macrogen Europe (The Netherlands). Sequences were edited using BioEdit software [38]. Sequence types (ST) and clonal complexes (CC) were determined by referring the obtained data onto the PubMLST website (https://pubmlst.org/), (accessed on 1 July 2022), as described in [32].

## Figures and Tables

**Table 1:** Results of *Campylobacter* spp. isolation from different shellfish species and sampling locations in Istria, Croatia.

Coast Location *		No. of Samples According Shelfish Species			Total of Samples	No. of Positive Samples		
		Oysters	Scallops	Mussels		Total	with *C. jejuni*	with *C. lari*
East	L-III	-	-	13	13	1	0	1
	L-V	-	-	13	13	2	0	2
West	L-I	-	-	13	13	7	0	7
	L-II	-	-	13	13	1	0	1
	L-IV	-	-	13	13	5	0	5
	L-VI	-	-	13	13	4	1	3
	L-VII	-	10	-	10	0	0	0
	L-VIII	-	10	-	10	0	0	0
	L-IX	3	7	-	10	0	0	0
**Total**		**3**	**27**	**78**	**108**	**20**	**1**	**19**

* see Figure 1 for location on the coast of Croatia.

**Table 2:** Results of *Campylobacter* species isolation from mussels by month and date of sampling, sea temperature and sampling locations in Istria, Croatia.

Month	Date of Sampling	Sea Temperature (°C)	Coast	Location	*Campylobacter* Species
August	2 August.	26	West	L-I	*C. lari*
	9 August	26		L-IV	*C. lari*
	9 August.	25		L-VI	*C. lari*
	16 August	25		L-I	*C. lari*
	16 August	27		L-IV	*C. lari*
	23 August	26		L-IV	*C. lari*
September	6 September	25	West	L-I	*C. lari*
	6 September	24		L-IV	*C. lari*
	13 September	25		L-I	*C. lari*
	13 September	25		L-VI	*C. lari*
	20 September	23		L-VI	*C. jejuni*
	27 September	23		L-VI	*C. lari*
October	4 October	23	West	L-I	*C. lari*
	4 October	21		L-II	*C. lari*
	13 October	20		L-IV	*C. lari*
	11 October	22		L-I	*C. lari*
	25 October	18		L-I	*C. lari*
	4 October	21	East	L-V	*C. lari*
	4 October	19		L-III	*C. lari*
	11 October	19		L-V	*C. lari*

**Table 3:** List of sequence types (ST) of *Campylobacter lari* isolates.

Isolate	ST	*adk*	*atpA*	*glnA*	*glyA*	*pgi*	*pgm*	*tkt*
sh01	**300**	7	1	1	53	1	84	2
sh03	223	128	6	1	1	1	1	36
sh04	223	128	6	1	1	1	1	36
sh05	**301**	**147**	150	31	88	129	**173**	149
sh06	**301**	**147**	150	31	88	129	**173**	149
sh09	**302**	109	113	94	27	**188**	141	146
sh11	**303**	103	4	1	1	1	3	36
sh12	76	7	1	1	1	1	1	2
sh13	77	7	1	1	53	1	3	2
sh14	**301**	**147**	150	31	88	129	**173**	149
sh17	77	7	1	1	53	1	3	2
sh18	82	8	6	1	1	1	1	86
sh19	**307**	8	2	1	2	**186**	1	2
sh20	**307**	8	2	1	2	**186**	1	2
sh21	**304**	20	39	14	49	41	35	20
sh22	**308**	20	18	**122**	49	**187**	14	**164**
sh23	**308**	20	18	**122**	49	**187**	14	**164**
sh25	**305**	17	114	93	27	73	136	101
sh26	**311**	30	**153**	95	104	**189**	**174**	125

Newly described alleles and STs are printed in **bold**.
